# Factors requiring adaptive re-planning in carbon ion radiotherapy for head and neck cancers

**DOI:** 10.3389/fonc.2026.1543304

**Published:** 2026-03-17

**Authors:** Takahiro Yamada, Naoko Okano, Akihiko Matsumura, Makoto Sakai, Hirofumi Shimada, Atsushi Musha, Tatsuya Ohno

**Affiliations:** 1 Organization for Promotion of Heavy Ion Medicine, Gunma University, Maebashi, Gunma, Japan; 2 Electromagnetic Application Systems Research Department, Research & Development Group, Hitachi Ltd., Hitachi, Ibaraki, Japan; 3 Gunma University Heavy Ion Medical Center, Maebashi, Gunma, Japan

**Keywords:** adaptive radiotherapy, broad beam, carbon ion radiotherapy, head and neck cancers, re-planning

## Abstract

**Background:**

The anatomical changes during carbon ion radiotherapy (CIRT) for head and neck cancers frequently require plan adaptations with adaptive re-planning. However, the details of the anatomical changes and their rates are not clear. The purpose of this study was to retrospectively evaluate the factors that require re-planning during CIRT for head and neck cancers.

**Methods:**

Thirty-seven patients who underwent 146 computed tomography (CT) scans and CIRT for head and neck cancers were evaluated retrospectively. To evaluate the re-planning situation, the frequency of the plan adaptation, reasons for plan adaptations, and causes of dose-distribution distortion were evaluated. Dose distributions were evaluated for two cases in which re-planning was carried out due to changes in tumor size and density on the beam path.

**Results:**

The main cause for dose-distribution distortion was a change in tumor size for 11 of 13 patients who underwent plan adaptation. Of the tumor-size changes, 64% were due to tumor expansion. For two patients, adaptive re-planning on weekly CT images resulted in a dose distribution equivalent to the original treatment plan in terms of target coverage and sparing of organs at risk.

**Conclusion:**

In CIRT for head and neck cancers, the main cause of dose-distribution distortion requiring re-planning was an increase in tumor size soon after treatment initiation.

## 1 Introduction

For head and neck cancers, anatomical changes, such as weight loss and tumor shrinkage, during a radiotherapy period have been reported (1). The anatomical changes around a treatment site may cause dose-distribution distortion, such as dose reduction to a target and dose increase to normal tissues. Various adaptive treatment strategies have been reported for X-ray intensity-modulated radiation therapy (IMRT) and proton therapy (1–6).

Valuable local control rates and safety have been reported in carbon ion radiotherapy (CIRT) for head and neck cancers due to superior dose distribution on the basis of physical properties and a cell-killing effect on the basis of biological properties (7–10). On the other hand, the good dose distribution implies the possibility of robustness reduction due to anatomical changes with electron density changes in the beam path.

At the Gunma University Heavy Ion Medical Center (GHMC), anatomical changes with weekly computed tomography (CT) has been evaluated during CIRT for head and neck cancers, and off-line re-planning was carried out if unintended variation in the dose distribution was observed. However, detailed information regarding the incidence of anatomical changes, the reason for re-planning, and the impact on dose-volume histogram parameters remain unclear. By clarifying these details, more reliable treatment will be able to be implemented by taking countermeasures against anatomical changes likely to occur during treatment. The purpose of this study was to quantify the anatomical variations specific to CIRT for head and neck cancers. The factors that require re-planning during CIRT were retrospectively evaluated.

## 2 Methods

### 2.1 Patient characteristics and treatment plans

Thirty-seven patients undergoing 146 CT scans during CIRT for head and neck cancers at GHMC in 2020 were evaluated retrospectively. For CIRT, patients were immobilized on a treatment couch using thermoplastic masks and vacuum bags. Treatment plans were generated using the treatment planning software Xio-N (Elekta, Stockholm, Sweden) with a two-step broad-beam CIRT, in which different target settings were used for the first nine fractions and the remaining fractions. The treatment planning method used in this study has been described in detail previously (7–9). Delineation of the gross tumor volume (GTV) was based on contrast-enhanced MRI. The clinical target volume (CTV) had at least a 5-mm margin around the GTV. CTV1 included all anatomic sites where the tumors were located, whereas CTV2 was limited to around the GTV. CTV1 is a target volume of the first nine fractions, and CTV2 is a target volume of the remaining fractions contoured by omitting the planning organ at risk volume (PRV) from CTV1. PTV1 and PTV2 had 2-mm margins around CTV1 and CTV2, respectively. The CTV and PTV margins were modified as necessary when the targets were close to the organs at risk. The radiation dose was prescribed at the isocenter of the PTVs. The PTVs were encompassed by the 95% isodose line of the prescribed dose. The dose fractions were 12 or 16. Total dose ranged from 52.8 to 70.4 Gy(RBE). Four fractions were irradiated per week, and weekly CT images were acquired the day before the first fraction and at fractions 5, 9, and 13. Both the simulation CT for treatment planning and the weekly CT scans were acquired as non-contrast CT images.

The dose evaluation process for re-planning was performed as follows. First, the dose distribution for each beam was calculated on the weekly CT images based on the initial plan. Second, the cumulative dose was estimated in two steps. The dose distribution that had already been delivered at the time of weekly CT acquisition was estimated by assuming that all prior fractions had been administered according to the treatment plan. This was then combined with the projected dose for the remaining fractions, assuming delivery with the beams calculated the remaining in the previous step, using Xio-N and MIM maestro (MIM Software Inc., OH). Third, if the doses to OARs or targets exceeded the tolerances (11), the beams were re-planned on the weekly CT to preserve the original planning concept. The specific dose constraints for CTV and OARs used in this study are summarized in **Supplementary Table 1**. This procedure was iteratively repeated until all dose constraints were satisfied.

### 2.2 Evaluation of actual re-planning

To evaluate re-planning, all patients were categorized as (A) not re-planned, (B) re-planned, or (C) plan adaptation by changing the fraction number of irradiation fields. For group (A), original plans were delivered throughout a whole treatment period. Instead of an irradiation field with dose-distribution distortion on weekly CT, an irradiation field with little change in dose distribution was delivered for patients in group (C). For groups (B) and (C), the dose distribution of each irradiation field was recalculated on the weekly CT and classified into four groups: (a) not re-planned, (b) re-planned, (c) plan adaptation by changing the fraction number of irradiation fields, and (d) not re-planned but dose distribution deteriorated. The detailed classification workflow for re-planning is illustrated in **Supplementary Figure 1**. The operational procedure for fraction rearrangement, including how irradiation fields were reassigned when dose distortion was detected on weekly CT, is illustrated in **Supplementary Figure 2**. The proportions of patients belonging to each category were calculated, and their 95% confidence intervals (CIs) were estimated using the Wilson score method.

The reasons for plan adaptations and the causes of the dose-distribution distortion were evaluated by an experienced radiation oncologist for dose distributions categorized as (b) and (c).

### 2.3 Evaluation of dose distributions

Dose distributions were evaluated for two cases in which re-planning was carried out due to changes in tumor size and density on the beam path. Three types of dose distributions were evaluated for each patient: (1) treatment plan, (2) dose distribution when irradiated as scheduled without plan adaptation, and (3) actual dose distribution with re-planning. For types (2) and (3), the dose distribution of each dose fraction was recalculated on the weekly CTs rigidly registered to the simulation CT, and summed for all fractions by deforming dose distributions on the basis of the anatomy in the planning CT. Deformable image registration (DIR) was conducted using the intensity-based DIR algorithm in MIM maestro. Maximum dose, minimum dose, and the volume percentage receiving 95% of prescribed dose (V95) were evaluated for the clinical target volume (CTV1) and CTV2. For organs at risk, the maximum doses in brainstems and optic nerves that were considered during treatment planning were evaluated in comparison with dose-volume constraints (11).

## 3 Results

The characteristics of patients and treatment plans are shown in **Table 1**. The incidence of re-planning was 80% (4 of 5 patients) for sarcoma, which was higher than that for non-squamous cell carcinoma (5 of 24 patients: 21%). **Table 2** shows the number of re-planned patients. Re-planning was performed for 11 patients (30%), and the fraction number of irradiation fields was changed for 2 patients (5%). Regarding the evaluation based on the number of CT simulations, 28 (54%) of patients were classified into groups (b), (c), or (d). In other words, the dose distribution deteriorated in about half the weekly CTs for patients who underwent re-planning.

**Table 1:** Characteristics of patients and treatment plans.

Characteristics	Number of patients	Number of re-planned patients
Diagnosis		
Non-squamous cell carcinoma	24	5
Sarcoma	5	4
Malignant melanoma	4	2
Other	4	2
Site		
Nasal cavity/Paranasal Sinus	18	6
Oral cavity	5	2
Parotid gland	5	0
Pharynx	2	2
Other	7	3
Dose fraction		
64.0 Gy(RBE)/16 fractions	24	9
57.6 Gy(RBE)/16 fractions	8	2
70.4 Gy(RBE)/16 fractions	3	2
57.6 Gy(RBE)/12 fractions	1	0
52.8 Gy(RBE)/12 fractions	1	0

**Table 2:** Summary of re-planned patients.

Characteristics	Number of patients (%)
Evaluation based on patient number	
(A) Not re-planned	24 (65)
(B) Re-planned	11 (30)
(C) Fraction rearrangement	2 (5)
Evaluation based on CT simulation number	
(a) Not re-planned	24 (46)
(b) Re-planned	14 (27)
(c) Fraction rearrangement	2 (4)
(d) Not re-planned, but dose distribution deteriorated	12 (23)
(b), (c) or (d)	28 (54)

**Figure 1:**
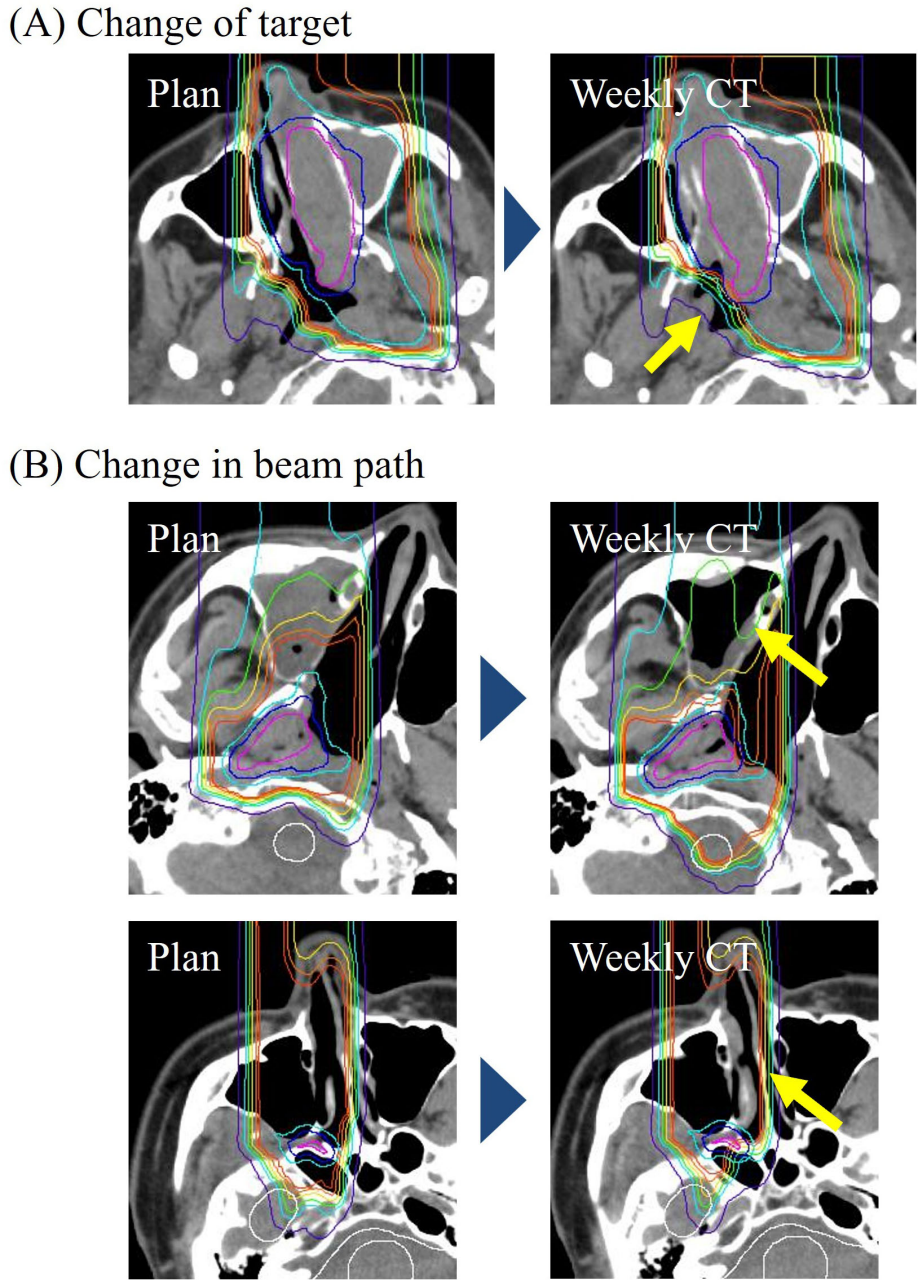
Anatomical change in CIRT for head and neck cancers. **(A)** shows an example of a change of target. In this example, the tumor expanded in the nasal cavity (yellow arrow). **(B)** shows examples of a change in beam path. The volume of filling in the maxillary sinuses and nasal cavity changed (yellow arrow).

The reasons for plan adaptation and causes of dose-distribution distortion are listed in **Table 3**. The main reason for plan adaptation was dose reduction in CTV (14 of 16 patients underwent plan adaptation: 88%). The main cause for the dose-distribution distortion was a change in tumor size (11 of 16 patients underwent plan adaptation: 69%), as shown in **Figure 1A**. Of the tumor-size changes, 64% were due to tumor growth. Changes in beam path, such as changes in the volume of filling in the maxillary sinuses and nasal cavity, as shown in **Figure 1B**, were observed in 5 of 16 patients who underwent plan adaptation (31%). Tumor width increased approximately 2–4 mm. Changes in beam path of several centimeters in water-equivalent thickness occurred in a part of the irradiated field.

**Table 3:** Plan adaptation reasons and causes of dose-distribution distortion.

Causes Reasons	Change of target		Change in beam path
	Expansion	Shrinkage	
Dose reduction in CTV	7	2	5
Dose increase in OARs	0	2	0

**Figure 2:**
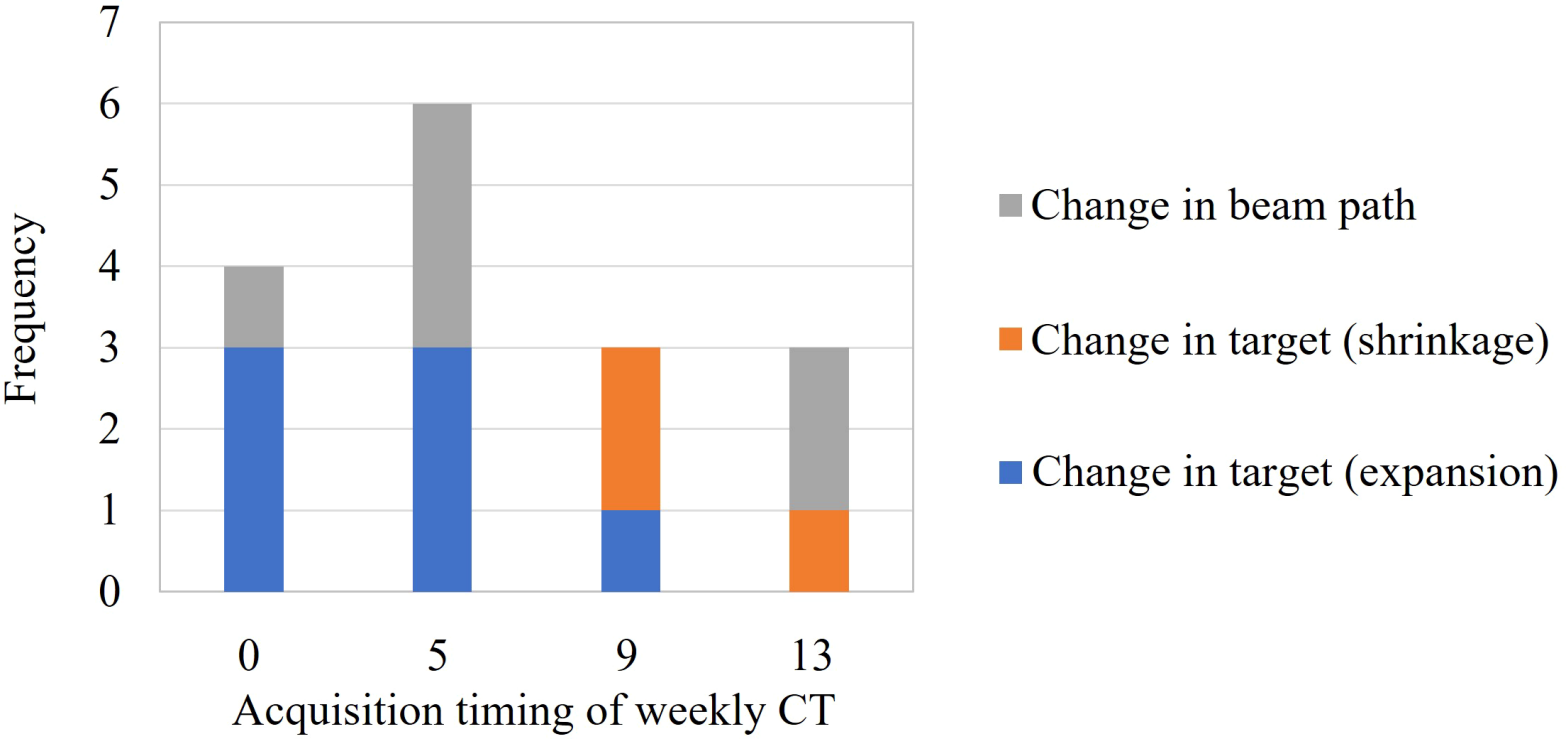
Causes of dose-distribution distortion.

**Figure 2** shows the causes of dose-distribution distortion classified by the acquisition timing of the weekly CT.

The target expansion was observed for about 2 weeks after the start of irradiation, and the target shrinkage was observed after the third week. Changes in beam path occurred regardless of the treatment duration.

**Figure 3:**
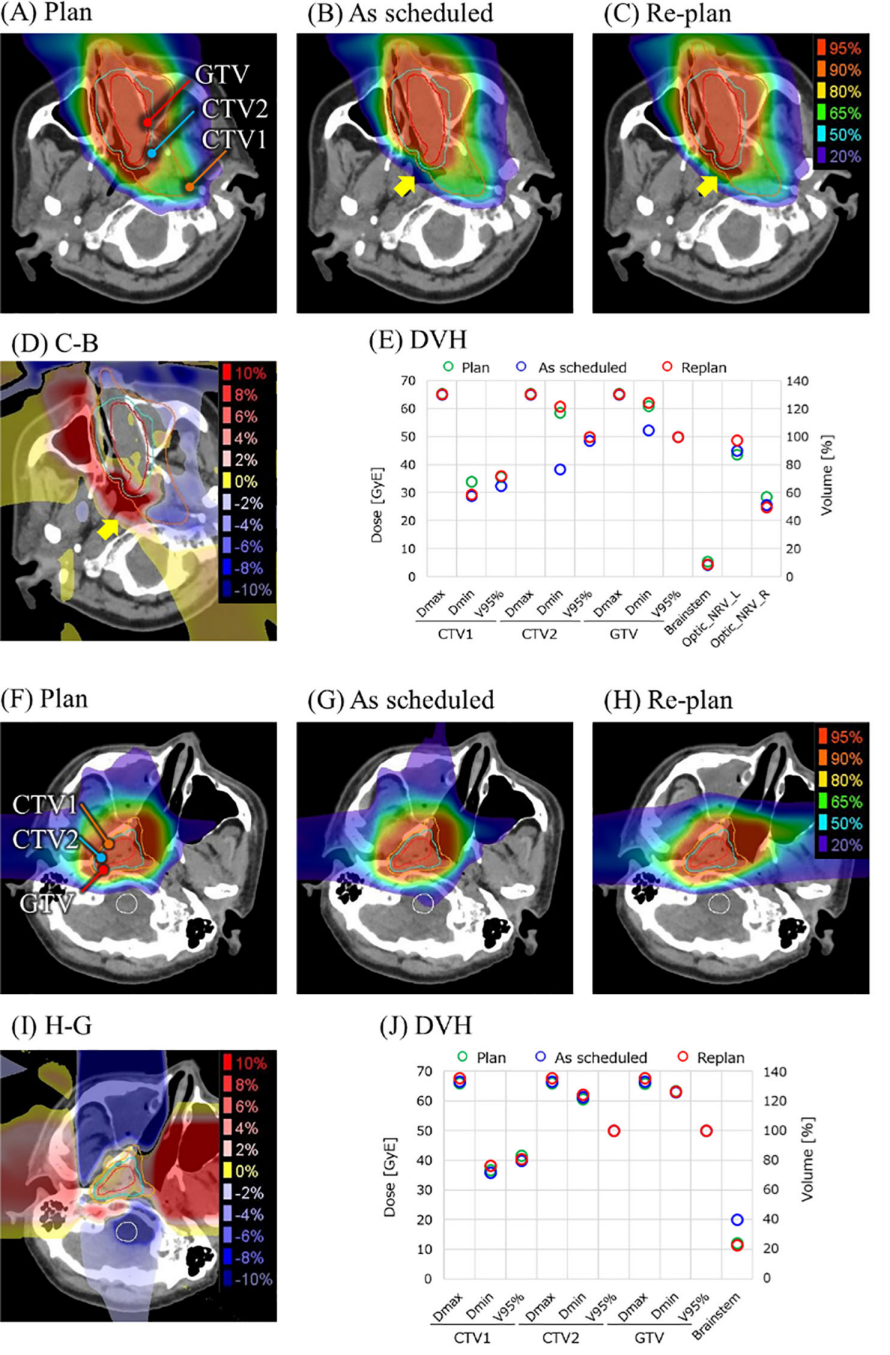
Change in dose distribution induced by anatomical changes. **(A–E)** are for a patient with a change in target shown in **Figure 1A**. **(F–J)** are for a patient with a change in beam path shown in **Figure 1B**. For these patients, dose distribution for the original treatment plan, dose distribution when irradiated as scheduled without plan adaptation, actual dose distribution with re-planning, dose-distribution distortion between plan and re-planning, and DVH parameters for targets and organs at risk (OARs) are shown. Dax, maximum dose; Dmin, minimum dose; GTV, gross tumor volume.

The variation in dose distribution and dose–volume histogram (DVH) indexes for the patients in **Figures 1A, B** are shown in **Figure 3**. **Figures 3A–E** are for a patient with the target expansion where the target coverage was improved by re-planning. The minimum dose of CTV2 was increased by approximately 20 Gy (RBE) from as scheduled to the same level as the treatment plan.

**Figures 3F–J** are for a patient with changes in beam path where the dose to the brainstem was reduced by re-planning. In addition to the re-planning, the fraction number of irradiation fields was changed so that the dose upstream on the target increased in the horizontal direction, as shown in **Figure 3I**.

## 4 Discussion

The re-planning of CIRT for head and neck cancers was retrospectively evaluated. Thirteen (40%) of 37 patients underwent plan adaptations, including re-planning and changing of the fraction number of irradiation fields. For patients who underwent re-planning, the dose distribution deteriorated in about half the weekly CTs.

The main cause of the dose-distribution distortion was a change in tumor size, of which an increase was dominant. As shown in **Figures 3A–E**, an increase in tumor size led to a decrease in the dose delivered to the target. Insufficient target dose may reduce the possibility of local control of the tumor.

The plan library method has been proposed as one solution for anatomical changes during treatment. In our study, tumor sizes increased in the nasal cavities of several patients, and the maximum incremental width was 2–4 millimeters. This intranasal increase was considered amenable to the plan library method (12–14), which has been reported for several treatment sites. In the plan library method, multiple treatment plans are generated in accordance with expected anatomical changes in advance, and an appropriate treatment plan is selected in accordance with the anatomy of the treatment day. In CIRT for head and neck cancers, in addition to the usual treatment plan, treatment plans in which the target is enlarged by several millimeters in the nasal cavity are generated in advance. With this plan library method, the anatomical change can be addressed without online re-planning even in broad-beam CIRT, which requires patient-specific tools such as a range compensator and collimator.

Another cause of the dose-distribution distortion was changes in beam path, such as changes in the volume of filling in the maxillary sinuses and nasal cavity. In **Figures 3F–J**, a change in cavity filling led to an increase in the dose to the brainstem, resulting in the necessity for re-planning. An increase in brainstem dose leads to increased risk of brain necrosis and cranial nerve damage. Changes in beam path cause a change in water-equivalent thickness of several centimeters in a portion of the irradiated field, which is difficult to respond to with the plan library method, which predicts the change in advance.

Several IMRT studies have reported anatomical changes during the treatment period of head and neck (15–17). While the tumor tends to shrink during the entire treatment period, some patients experience tumor growth within 1–2 weeks after starting treatment (15). The causes of dose-distribution distortion evaluated in our study indicate a change from enlargement to shrinkage after the first two weeks of treatment.

Hu et al. reported that the percentage of patients who underwent re-planning of proton beam therapy for head and neck cancers was 49% (18), which is comparable to that in our study (35%). Hu et al. treated patients with intensity-modulated proton therapy irradiation, which is considered more sensitive to anatomical changes than the broad-beam CIRT carried out in our study. There might be differences in eligible patients between proton therapy and CIRT, and it is difficult to compare two treatment groups because our patients mainly consisted of non-squamous cell carcinoma without lymph node metastasis, and irradiated target volume was smaller than that in the proton therapy population.

This study shows that changes in tumor size and changes in the beam path such as changes in the volume of filling in the maxillary sinuses and nasal cavity causes a reduction in target dose or an increase in the dose to OARs. Particularly in CIRT for head and neck cancers, to implement the initially planned treatment as scheduled, it is essential to acquire CT images to accurately assess the patient’s internal condition and adjust the treatment plan as much as possible to adapt to that condition.

The present study includes the following limitations. First, the evaluation in our study was based on weekly CTs. For a more detailed evaluation, daily CTs should preferably be used. However, Bobić et al. reported that the variation in dose distribution due to the difference in adaptation frequency between weekly and daily is small for head and neck IMPT (19). This seems to be the same for CIRT, so an evaluation based on higher frequency imaging would not significantly change the conclusions of this study.

Second, the number of patients was relatively small. Further comparative clinical data are needed.

## 5 Conclusion

In carbon ion radiotherapy (CIRT) for head and neck cancers, the main cause of dose-distribution distortion requiring re-planning was an increase in tumor size soon after treatment initiation. Specifically, 69% of dose-distribution distortions were due to changes in tumor size, of which 64% were due to tumor expansion. The tumor growth in the nasal cavity was considered responsive to the plan library method, with which an appropriate treatment plan was selected from those prepared in advance on the basis of predictions in tumor-size changes.

## Data Availability

The original contributions presented in the study are included in the article/**Supplementary Material**. Further inquiries can be directed to the corresponding author.
