# Supplementary figures and images for: Factors requiring adaptive re-planning in carbon ion radiotherapy for head and neck cancers
Source: Front Oncol. 2026 Mar 17;16:1543304. doi: 10.3389/fonc.2026.1543304 (PMC13035771; doi:10.3389/fonc.2026.1543304)

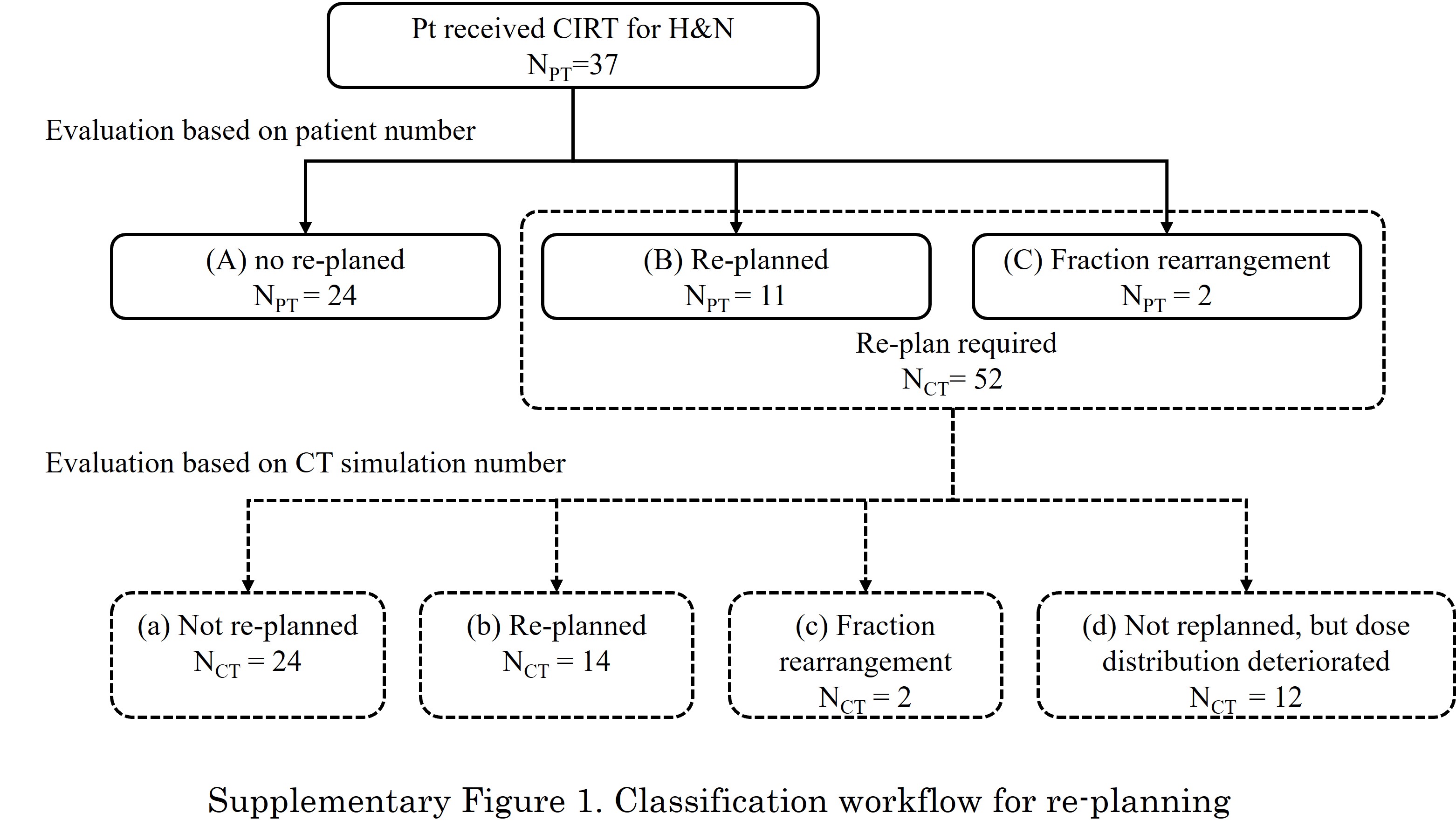

Supplement: Supplementary file 1 [file Image1.jpg]

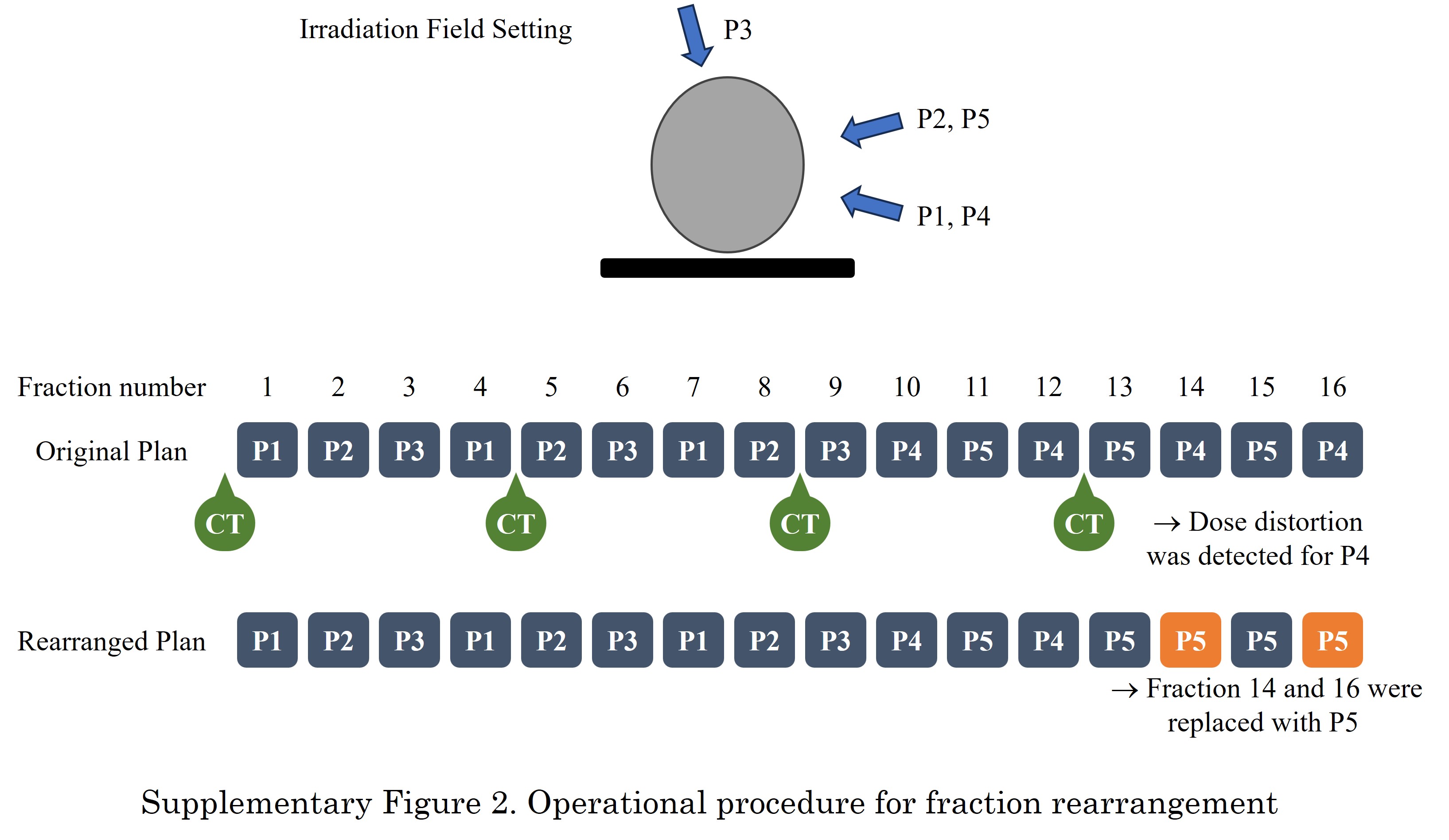

Supplement: Supplementary file 2 [file Image2.jpg]

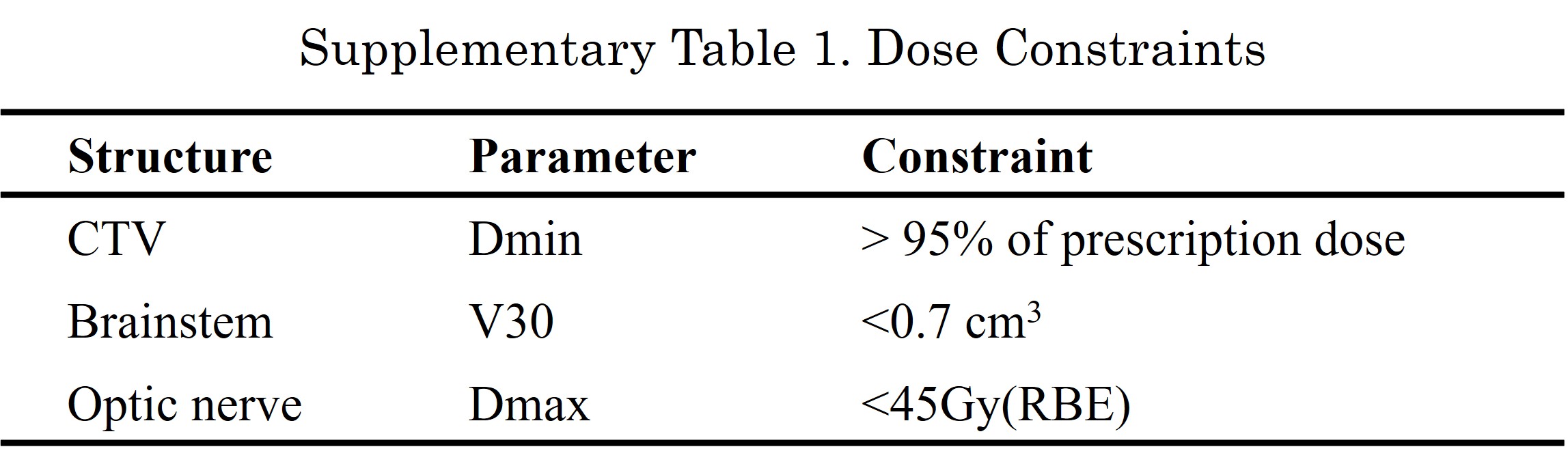

Supplement: Supplementary file 3 [file Supplementaryfile1.jpg]
